# Quercetin: Its Antioxidant Mechanism, Antibacterial Properties and Potential Application in Prevention and Control of Toxipathy

**DOI:** 10.3390/molecules27196545

**Published:** 2022-10-03

**Authors:** Weidong Qi, Wanxiang Qi, Dongwei Xiong, Miao Long

**Affiliations:** Key Laboratory of Livestock Infectious Diseases, Ministry of Education, College of Animal Science and Veterinary Medicine, Shenyang Agricultural University, Shenyang 110866, China

**Keywords:** quercetin, antioxidant, toxipathy

## Abstract

Quercetin, as a flavonol compound found in plants, has a variety of biological activities. It is widely present in nature and the human diet, with powerful oxidative properties and biological activities. In this review, the antioxidant mechanism and broad-spectrum antibacterial properties of quercetin are revealed; the intervention effects of quercetin on pesticide poisoning and the pathway of action are investigated; the toxic effects of main mycotoxins on the collection and the detoxification process of quercetin are summarized; whether it is able to reduce the toxicity of mycotoxins is proved; and the harmful effects of heavy metal poisoning on the collection, the prevention, and control of quercetin are evaluated. This review is expected to enrich the understanding of the properties of quercetin and promote its better application in clinical practice.

## 1. Introduction

**Figure 1:**
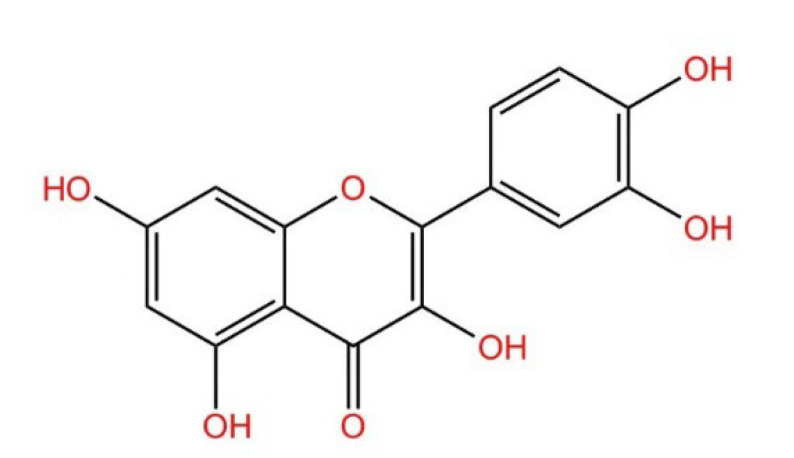
Structural formula of quercetin.

During the last few decades, medicinal plants have gained wide popularity due to their low incidence, mildness of side effects, low price, and natural origin, among which quercetin is one of the well-known types of plant metabolites [1]. Quercetin is a flavonoid widely found in vegetables and fruits. Its name comes from Quercetum (oak forest), used since 1857, its molecular formula is C_15_H_10_O_7_; its chemical structure formula (Figure 1) has unique biological properties, can improve physical and mental status, and reduce viral infection [2,3]. It is a naturally occurring acute coenzyme transport inhibitor [4]. Quercetin exists in its various glycoside forms and the five yellow compounds isolated from dietary quercetin (e.g., quercetin-3-glucoside or isoquercitrin, quercetin-4′-glucoside, quercetin-3,4′-diglucoside [5]). These five compounds are quercetin 3-O-beta-D-glucopyranoside (DA), kaempferol 3-O-(6″-trans-coumarin)-beta-D-glucopyranoside (D1), kaempferol 3-O-(2″,4″-diacetyl-p-coumaroyl-6″-trans-coumaroyl)-beta-D-glucopyranoside (A), kaempferol 3-O-(2″-6″-di-trans-p-coumaroyl)-beta-D-glucopyranoside (D7), and kaempferol 3-O-beta-D-glucopyranoside (B). Quercetin contains multiple hydroxyl groups and its molecular structure comprises four reactive groups, i.e., dihydroxy group between the A ring; o-dihydroxy group B; and C2 and C3 double bonds of the C ring, and the 4-carbonyl group has physiological activities that readily undergo esterification with carboxyl groups and anti-cardiovascular disease [6]. The antioxidant and anti-inflammatory properties of quercetin are closely related to the prevention and treatment of pesticide poisoning and heavy metal poisoning. In addition, quercetin plays an essential role in reducing mycotoxins and protecting cells from damage [3]. However, there are few studies related to the blocking effects of quercetin on various toxic diseases. This review aims to summarize and analyze the preventative effects of quercetin on heavy metal poisoning, pesticide poisoning, mycotoxin poisoning, and inflammation caused by toxic diseases so as to provide a scientific basis for its better clinical application.

## 2. Main Pharmacological Activities of Quercetin

### 2.1. Antioxidants

By studying the chemical structure of quercetin, the International Union of Pure and Applied Chemistry named quercetin as 3,3′,4′,5,7-pentahydroxyflavone, which indicates that quercetin has an OH group attached at positions 3, 5, 7, 3′, and 4′ [2]. The antioxidant mechanism of quercetin in vivo is mainly reflected in its effects on glutathione (GSH), signal transduction pathways, reactive oxygen species (ROS), and enzyme activities. The antioxidant properties of quercetin show a concentration dependence in the low dose range but too much of the antioxidant brings about the opposite result [7].

#### 2.1.1. Quercetin Achieves Antioxidant Effects by Affecting GSH as a Reactive Hydrogen Donor

Quercetin can enhance the antioxidant capacity of the body by regulating the level of GSH. This is because free radicals are produced by the body during metabolic processes, which cause genetic mutations and cell membrane damage; induce various diseases, such as heart disease, liver disease, and diabetes; and accelerates aging of the body [8,9]. Once the body produces free radicals, superoxide dismutase (SOD) will quickly convert O_2_^−^ to H_2_O_2_ and will be further catalyzed into non-toxic H_2_O and GSH. As the reaction hydrogen donor, the level of GSH determines the rate of the reaction [10]. In addition, one study found that the application of quercetin treatment in renal ischemia/reperfusion (I/R) increased the level of GSH [11]. The ability of quercetin to induce GSH synthesis was also confirmed in subsequent studies. Gao et al. [12] reported the inhibitory effect of quercetin on GSH at the 0.5% level and found that GSH reductase can catalyze the reduction reaction of GSSG in erythrocytes and liver, leading to the formation of GSH, whereas while high doses of quercetin can be used to regulate GSH, dynamic balance is affected by the action of peroxidase; H_2_O_2_ is converted to H_2_O and GSH is oxidized to oxidized glutathione disulfide, due to the aforementioned reactions resulting in a dynamic balance of GSH. This may lead to the suppression of the level of GSH at low doses.

#### 2.1.2. Quercetin Achieves Antioxidant Effects through Positive Effects on Various Signal Transduction Pathways

It was found that quercetin counteracts atherosclerosis by reversing the increased expression of NADPH oxidase in oxLDL and the oxLDL-induced decreases in activation of AMP-activated protein kinase, thereby inhibiting NF-κB signaling and holding AKT/eNOS function [13]. In addition, Granado-Serrano et al. [10] found that quercetin improves the antioxidant capacity of cells by activating the intracellular p38 MAPK pathway, increasing intracellular GSH levels and providing a source of hydrogen donors in the scavenging of free radical reactions. It is known that adverse environmental factors increase the production of ROS. These factors increase the activity of mitochondrial electron transport chains, which is an important source of intracellular ROS production [14]. The body fights against free radicals through two main defense systems: non-enzymatic antioxidants represented by vitamins and trace elements (such as vitamin C, vitamin E, selenium, copper, manganese, etc.) and enzymatic antioxidants represented by SOD, including catalase, glutathionase, etc. Quercetin can promote the antioxidant defense system and maintain oxidative homeostasis not only by regulating the non-enzyme-dependent antioxidant defense system and enzyme-mediated antioxidant defense system but also by regulating MAPK, NRFB, AMPK, and other signaling pathways induced by ROS [15,16,17,18,19].

By affecting signal transduction pathways, quercetin can modulate enzymes or antioxidant substances and enhance antioxidant properties, thereby preventing disease progression. In psoriasis, quercetin was found to promote the disease recovery by up-regulating the expression of TNF receptor-associated factor 3 and down-regulating the expression of NF-κB, inducing kinase. In addition, quercetin achieves protection against acute spinal cord injury by up-regulating the activity of SOD, down-regulating the level of malondialdehyde (MDA), and inhibiting the p38MAPK/iNOS signaling pathway [20]. In addition, quercetin reduces imiquimod (IMQ)-induced MDA levels in skin tissues and enhances catalase, SOD, and GSH activities, which together improve the antioxidant properties of the body [21].

It was found that quercetin improves antioxidant capacity by modulating signaling pathways. For example, quercetin promotes the functional recovery of mobile mediators after cerebral ischemia by promoting antioxidant signaling, translating the TGFβ-2/PI3K/AKT pathway, and increasing resistance to apoptosis. It also controls the development of atherosclerosis induced by high fructose diet by enhancing PI3K/AKT and inhibiting ROS [22,23,24].

#### 2.1.3. Quercetin Prevents Antioxidant Damage by Eliminating ROS

It is known that quercetin can scavenge ROS, and most of the oxidative damage in vivo is attributed to ROS, so quercetin can resist oxidative damage, such as radiation-induced ultraviolet radiation B (UVB) skin lesions, respiratory damage, and other oxidative damage diseases [25]. Human skin is highly functional and can withstand many types of environmental damage; however, UVB induces an imbalance of endogenous antioxidant systems and a transient increase in ROS, which increases the level of inflammation and free radicals and affects cellular processes. Studies have shown that quercetin not only prevents UVB radiation damage by reducing ROS-induced damage to mitochondria but also by scavenging ROS, in addition to inhibiting mitochondrial membrane depolarization and cell membrane movement. Thus, it seems that quercetin can prevent UVB-induced skin damage by suppressing this imbalance [26,27].

In addition, exposure to ambient fine particulate matter (PM2.5) was found to cause respiratory disease, resulting in respiratory damage and a range of adverse changes, such as increased ROS production, suppressed mitochondrial expression, and weakened 16HBE cell activity. Quercetin may stimulate 16HBE cells to repair oxidative damage after PM2.5 exposure through the anti-inflammatory process and the production of ROS [28].

Quercetin enhances antioxidant activity and inhibits lipid cultivation, and it is effective in the treatment of oxidative liver damage [29]. Quercetin was found to restore endogenous redox homeostasis not only by scavenging free radicals and increasing the level of GSH but also by directly scavenging ROS and hydroxyl radicals under hypoxia. The reduction of oxidative stress-induced neurodegeneration in the hippocampal region thereby reversed hypoxia-induced memory impairment [30].

It was found that ionizing radiation induces this type of damage through increased cellular damage or increased free radical formation and cell death due to ROS, whereas bioflavonoids, as a redox agent, can inhibit the toxicity of free radicals and enhance antioxidant properties in vivo. Quercetin protects cells from radiation and genotoxicity-induced damage by increasing endogenous antioxidant and scavenging free radical levels [31,32,33].

On the other hand, quercetin can prevent oxidant damage by inhibiting oxidative stress. Oxidative stress is caused by an imbalance between antioxidants and oxidants in the body, and the reaction process tends to be oxidized, which, once oxidized, leads to the secretion of high protein enzymes and inflammatory infiltration of neutrophils. In contrast, quercetin can regulate the balance between antioxidants and oxidants to suppress oxidative stress. Various experimental studies demonstrated that quercetin inhibited acrylamide-induced oxidative damage in rats, radiation-induced brain damage in rats, cadmium fluoride-induced neurodegenerative disease and oxidative stress, and diabetes-induced nerve damage in the rat retina. It protects nerves, the brain, or other cells of the body from damage caused by oxidation by regulating the level of antioxidants [34,35,36].

It was found that quercetin can modulate the expression of antioxidant-related genes in A549 cells to alleviate oxidative stress. In addition, quercetin can increase the level of GSH and decrease the level of ROS to prevent paraquat-induced oxidative damage [37]. Due to the strong inhibitory and scavenging effects of quercetin on ROS, it also protects sperm from the adverse effects of ROS and maintains the function of male germ cells [38]. As quercetin exerts protective effects on gastric epithelial GES-1 cells, it was able to inhibit ROS-induced damage to gastric epithelial cells caused by oxidants such as H_2_O_2_. In gastric epithelial cells, quercetin can prevent oxidative damage and inhibit ROS production during acute gastric mucosal injury in mice [39].

#### 2.1.4. Effect of Quercetin on Enzyme Activity

Previous research has shown that two enzymes, acetylcholinesterase and butyrylcholinesterase, are associated with oxidative properties, while the -OH group on the lateral benzene ring of quercetin binds to important amino acid residues in the active sites of both enzymes and inhibits their oxidative effects [40]. In addition, Odbayar et al. found that quercetin increases the activity of antioxidant enzymes, such as GSH transferase and the aldo-keto reductase [41].

Pre-treatment with quercetin was reported to significantly increase the levels of expression of endogenous antioxidant enzymes, such as GSH peroxidase, Cu/Zn SOD, Mn SOD, and peroxidase, in CA1 vertebral neurons of the hippocampus of animals suffering ischemic injury. This suggests that quercetin may be a potential neuroprotective agent against ischemia, which protects CA1 vertebral neurons from I/R injury in the hippocampal region of animals [42].

Quercetin has also been shown to prevent heart damage by scavenging anaerobic free radicals induced by lipopolysaccharide-induced endotoxemia. Lipopolysaccharide has been reported to induce biochemical and histopathological damage to the myocardium in the models of endotoxemia. In rat model experiments, rats treated with lipopolysaccharide exhibited decreases in catalase and SOD activities and a significant increase in MDA levels in heart tissue. In contrast, treatment with quercetin significantly reduced MDA levels and increased SOD and catalase levels. This finding indicated that quercetin enhanced the antioxidant defense system [43].

### 2.2. Antibacterial Properties

The antibacterial mechanisms of quercetin have been reported to include: altering the permeability of bacterial cells; disrupting bacterial cell walls; inhibiting the synthesis of nucleic acids, thereby affecting the synthesis and expression of proproteins; and reducing enzyme activity. The results indicated that quercetin has broad-spectrum antibacterial properties, and it exerts a good inhibitory effect not only on bacteria but also on fungi to a significant extent. Quercetin also shows a good inhibitory effect on the growth of pathogenic bacteria, such as *Salmonella enterica*, *Escherichia coli*, *Pseudomonas aeruginosa*, *Staphylococcus aureus*, and *Aspergillus* [44]. In addition, quercetin affects the growth of *E. coli* by altering the activity of adenosine triphosphate [45].

Furthermore, TEM images showed that the treatment with quercetin (50× minimum inhibitory concentration (MIC)) eventually caused *E. coli* to be cavitated and killed, and such treatment can disrupt the cell membrane and cell wall of *S. aureus* (10× MIC). In the same study by Hossion et al. [44], a novel artificially designed synthetic quercetin acyl glucoside was found to significantly inhibit the growth of *Pseudomonas aeruginosa*, *S. aureus*, and *E. coli*. In addition, the extract of poplar plum (containing quercetin) showed significant antibacterial activity against Listeria monocytogenes, *Salmonella* spp. and *Shigella* spp. with MIC values ranging from 2.07 to 8.28 mg/mL. Wang et al. [46] found that quercetin could protect rats from catheter-associated *S. aureus* infection by inhibiting the activity of thrombin. In addition, sugarcane bagasse (with 470 mg quercetin/g polyphenol) extract was found to inhibit the growth of *L. monocytogenes*, *S. aureus*, *E. coli*, and *Salmonella typhi* [47].

In addition, quercetin was found to suppress abiotic surface colonization genes of *L. monocytogenes* at concentrations below the MIC [48]. Qayyum et al. [49] found that quercetin (½ × MIC) remained inhibitory to *E. faecalis* MTCC 2729 at MIC (256 g/mL) under scanning electron microscopy and confocal laser scanning microscopy. A study by Wang et al. confirmed that quercetin can inhibit the formation of *Streptococcus hepatitis* biofilm. In addition, quercetin prevents bacterial adhesion, inhibits population intervention pathways, alters or disrupts plasma membranes, and inhibits efflux pumps, thereby preventing nucleic acid synthesis. A study by Lee et al. [50] discovered that quercetin has an inhibitory effect on genes related to bacterial adhesion.

## 3. Preventing Poisoning as an Application of Quercetin

### 3.1. Preventing of Mycotoxin Poisoning

Mycotoxins are toxic substances produced in many foods and are secondary metabolites synthesized by molds, which are one of the major causes of food spoilage and associated issues in various foodstuffs and fodder and act as a ubiquitous environmental pollutant [51,52,53]. As shown in Table 1, mycotoxins have significant toxic effects on tissues and organs of humans and animals.

#### 3.1.1. Prevention of Deoxynivalenol (DON) Poisoning

*Fusarium* spp. can cause significant yield losses, of which deterioration in quality through contamination of grains with fungal toxins, such as trichothecenes, is an important category of fungal toxin contamination and *Fusarium* spp. includes a wide variety of fungal pathogens [59]. Among the Fusarium toxins, DON poses the greatest risk to humans and livestock and is generally referred to as a vomitoxin [59,60]. The toxic effects of DON arise mainly via interference with the gastrointestinal and immune systems. Studies have shown that low doses of DON can only cause the irritation of the gastrointestinal tract and do not bring about significant clinical symptoms, whereas high doses of DON cause vomiting and esophageal gastric ulcers [61]. The toxicity of DON goes beyond this, as it also inhibits protein synthesis, and reduces tryptophan uptake by the brain and the neurotoxicity of DON in microglia [62,63]. Currently, the main method used to inhibit Fusarium is mainly through azole insecticides, but the disadvantages of this insecticide are also obvious; not only is the effect not very significant because of drug resistance and other reasons but also due to the long-term repeated use of dispersion and persistence in the environment [64,65,66]. Therefore, quercetin, which is not only novel and environmentally friendly but also biologically active, came to our awareness on account of its mechanisms of defense against abiotic and biotic stresses and environmental interactions [67,68]. It was shown that cells pretreated with quercetin (1 mM) showed better resistance to DON-induced lipid peroxidation, loss of mitochondrial membrane potential, ROS generation, cell cycle arrest, down-regulation of neuronal biomarkers, and DNA damage. In a study by Yang et al., it was shown that 15-acetyl deoxynivalenol (15ADON) accounts for a large proportion of the DON family and co-exists with the prototype DON. The results indicated that quercetin treatment largely restored the increased ROS levels and inhibited their growth rate. The results showed that quercetin treatment largely reduced ROS levels and inhibited their growth. Furthermore, Pomothy et al. studied the effects of quercetin on DON-exposed porcine intestinal epithelial cell lines [69]. Metabolomic analysis showed that quercetin plays an important role in the cytotoxic effects and protective functions induced by DON and 15ADON.

#### 3.1.2. Aflatoxin (AFT) Poisoning

*Aspergillus flavus* is a saprophytic filamentous fungus that contaminates both pre-harvest and post-harvest seed crops, producing the carcinogenic secondary metabolite aflatoxin [70], which is a mutagenic, teratogenic, and carcinogenic toxin in animals and humans [71,72]. To prevent the adverse effects produced by *Aspergillus flavus* and its metabolites, the addition of mold inhibitors is one of the important measures used, among which quercetin, a natural mold inhibitor, has become the primary choice due to its low impact on the environment and animal organism [73]. It has been shown that although quercetin does not affect phosphatidylserine externalization and nucleation in *Aspergillus flavus*, it reduces ROS levels and regulates the expression of development-related genes and AFT production-related genes to inhibit proliferation and AFT biosynthesis [74]. In a study by Siess et al. [75], the mechanism of anti-AFB1 initiation effect of quercetin was expounded, which included the enhancement of enzymes involved in AFB1 detoxification (GSH S-transferase, UDP-glucuronosyltransferase), the increased formation of AFB1-GSH conjugates, and the inhibition of AFB1 binding to DNA. Aflatoxin B1 (AFB1) accounts for a high proportion in *A. flavus* and AFB1 is the most toxic, and its toxic effects specifically include the induction of oxidative stress pathways, increase in lipid peroxidation, and decrease in the level of antioxidant enzymes [76]. Not only that, Tan et al. [77] confirmed that AFB1 binds to human serum albumin (HSA) with high affinity, but it was found that quercetin competes with AFB1 to bind HSA, and the binding constant of quercetin-HAS complex is significantly higher than that of the AFB1-HAS complex, and quercetin is able to remove part of HAS from AFB1 and reduce its binding fraction. Numerous studies have investigated the toxicity of AFB1 exposure in animals during embryonic life to their central and peripheral nervous systems. Previous research has implied that the exposure of AFB1 to rat offspring prenatally delays the development of motor activity, motor coordination, exploratory behavior, reflex responses, and learning abilities [78,79]. With increasing concentrations of exposed AFB1, histopathological alterations in the brain of rats may include the dilatation of the lateral ventricles, the contraction of white and grey matter, and the depletion of nerve fibers in the spinal cord [80]. Furthermore, a study by Gugliandolo et al. demonstrated that oral supplementation with quercetin increased SOD activity, glutathione peroxidase, and catalase levels in the brain and reduced lipid peroxidation in AFB1-treated mice. This was confirmed in a study by Choi [81], in which the effect of quercetin on AFB1-treated HepG2 cells was evaluated and the results showed that quercetin not only inhibited lipid peroxidation and promoted antioxidant defense systems but also suppressed ROS and cytotoxicity. Furthermore, a study by Ghadiri et al. [82] indicated that quercetin exerts its beneficial effects by inhibiting the biotransformation of AFB1 and counteracting its pro-oxidant effects. In addition, studies by Buening et al. and Guengerich et al. [83,84] suggested that quercetin inhibited cytochrome c (P-450) reductase in human liver microsomes in vitro sex-specific liver experiments.

#### 3.1.3. Preventing of Ochratoxin A (OTA) Poisoning

Ochratoxins are mainly produced by *Aspergillus* and *Penicillium* [85]. Studies have shown that OTA is one of the most important fungal toxin contaminants in a wide range of foods produced by molds of the genera *Penicillium* and *Aspergillus*. It has hepatotoxic, nephrotoxic, teratogenic, and immunosuppressive effects [86]. Studies have found that quercetin has inhibitory and mitigating effects on OTA-induced immunotoxicity [87]. Due to its widespread nature, OTA is present in various animal tissues, even in human blood and breast milk, adversely affecting the safety of humans [88]. OTA has been shown to disrupt the immune system by inhibiting the lipogenic component of bone marrow mesenchymal stem cells [89]. In contrast, the inhibitory effect of quercetin on adipocyte differentiation is dose-dependent and is accompanied by a decrease in ROS production [90]. A study by Abdelrahman et al. similarly confirmed that quercetin can attenuate OTA-induced immunotoxicity by activating the PI3K/AKT signaling pathway and ameliorating oxidative stress [87]. Furthermore, in a study by Romero et al. [91], by adding quercetin at concentrations of 250 mg/L and 500 mg/L sequentially to agar containing OTA, the former showed significant signs of reduction in the growth rate of OTA, while the latter completely inhibited its growth. OTA binds to plasma albumin with equally high affinity [92]. Quercetin is also known to bind to HSA [93] and reducing the binding sites of OTA on HAS accelerates its elimination and potentially decreases toxicity [94]. In the results of Poór et al. [95], among the 13 flavonoids tested in the experiment, galangin and quercetin are the most effective competitors of OTA. Furthermore, research by Ramyaa et al. [96] found that quercetin not only prevented OTA-induced apoptosis but also inhibited the activation of the caspase cascade that leads to DNA breakage.

#### 3.1.4. Prevention of Zearalenone (ZEN) Poisoning

ZEN is a fungal toxin produced by four species of Fusarium, namely *Fusarium graminearum*, *Fusarium* spp, *Fusarium sylvatica*, and *Fusarium cereals* [97,98]. ZEN is widely present in nature and many important crops are contaminated and animal bodies are contaminated by feeding on crops containing ZEN [99]. Different studies have shown that ZEN has toxic effects both in vivo and in vitro, and in vivo the main target organ of action is the reproductive organ, which can induce endoplasmic reticulum (ER) stress-mediated apoptosis in leukemic cells [100]. It also induces cytotoxicity through the production of ROS, which leads to apoptosis by lipid peroxidation, mitochondrial pathways, and DNA damage [101,102,103]. ER plays a crucial role in multicellular organism protein processes, including protein transport, protein folding, and intracellular calcium regulation. In contrast, ZEN leads to ER stress due to the disruption of the redox state, the accumulation of unfolded proteins and protein transport, which then further triggers the unfolded protein response [104]. The unfolded protein response is an adaptive response that restores ER homeostasis by activating the three proximal sensors TF6 (activating transcription factor 6), PKR-like endoplasmic reticulum kinase, and inositol-requiring enzyme 1α (IRE1α) under short and mild ER stress, but severe and prolonged ER stress activates downstream effectors, including JNK, CHOP (C/EBP homologous protein), and members of the cystathionine and Bcl2 families, leading to apoptosis [105,106]. The antioxidant properties of quercetin were shown to help combat ER stress and reduce ZEN-induced apoptosis [107]. In addition, in a study by Salem et al. [108,109], quercetin had a significant protective effect during the activation of ER stress and apoptosis by α- and β-zearalenol in HCT116 cells. Saffronin and quercetin protect HCT116 and HEK293 cells from ZEN-induced apoptosis by reducing ER stress.

### 3.2. Preventing Pesticide Poisoning

Pesticides are mostly used to control pests that harm plants, animals, and humans, and their application may cause harm to humans through occupational exposure or ingestion of contaminated food and water, which can result in a range of injuries, such as cardiotoxicity, once the pesticide enters the body [110]. Quercetin is one of the most effective drugs to improve cardiotoxic diseases caused by pesticides [110]. The harmful effects of pesticides, especially traditional pesticides, on pollinators and their residues in floral fragrances are of wide concern [111]. Conservative estimates have implied that approximately 258,234 people die globally each year from pesticide self-poisoning [112].

#### 3.2.1. Preventing Imidacloprid Poisoning

Imidacloprid, as a systemic insecticide belonging to the neonicotinoid family of insecticides, has been one of the main products used to control agricultural pests since its introduction in 1991 [113]. Imidacloprid can act as an agonist of acetylcholine receptors interfering with neuronal signaling and can cause paralysis, hyperstimulation, and death [114]. A study by Liu et al. found that the lifespan of *Apis cerana* workers exposed to long-term imidacloprid is significantly shortened, while the treatment of the aforementioned *Apis* *cerana* workers with appropriate amounts of quercetin prolonged their lifespan [115]. Imidacloprid exposure caused ROS accumulation, blocked the activity of antioxidant enzymes, and enhanced mitochondrial apoptosis, which can stimulate oxidative stress pathways to trigger apoptosis in grass carp hepatocytes. In contrast, quercetin counteracts these effects through the PTEN/PI3K/AKT pathway [116]. Ardalani et al. [117] used quercetin as one of the most abundant phytochemicals in plants to assess the up-regulatory effect of quercetin on the function of the honeybee interpretation system. The results indicated that the intake of quercetin led to a significant decrease in the concentration of imidacloprid in honeybees.

#### 3.2.2. Preventing Organophosphorus Pesticide Poisoning

Because the mammalian gestation process consists of two critical periods, the first of which is the formation of organs, organophosphorus pesticide poisoning is extremely damaging to the mammalian fetal brain. Among other things, the process of rapid brain growth is at this stage [118,119]. As a result, organophosphorus interferes with the growth and differentiation of many tissues (especially the brain) during fetal life, which thus affects cell proliferation and leads to brain dysfunction. Studies have shown that even low doses of organophosphorus can cause biochemical and neurobehavioral abnormalities. In contrast, a combined treatment with quercetin improved organophosphorus-induced developmental neurotoxicity by inhibiting oxidative stress and neurotransmission disturbances that can promote cellular redox status [120].

### 3.3. Preventing Heavy Metal Poisoning

Heavy metals cause the oxidative deterioration of biomolecules by initiating free radical-mediated chain reactions that lead to protein oxidation and the oxidation of DNA and RNA, lipid peroxidation, and quercetin as an antioxidant for heavy metal poisoning diseases intervenes both directly and indirectly. This not only directly chelates toxic metals and quenches free radicals to affect biological systems but also indirectly regenerates endogenous antioxidants to enhance cellular antioxidant defense mechanisms [121].

#### 3.3.1. Preventing Cadmium (Cd)-Induced Toxic Diseases

Cd is accumulative in the animal organism and is difficult to excrete after entering the organism, and its toxic effects are mainly exerted through oxidative stress causing slow growth and liver and kidney dysfunction [122]. The main mechanism of Cd-induced cytotoxicity is the regulation of cellular redox status by briefly increasing ROS levels and mitochondrial damage [123,124]. The application of Cd induces an increase in plasma-labeled enzyme activity, and although quercetin does not inhibit this process, it weakens the oxidative damage induced by Cd [125,126]. Since Cd-induced dysfunction relies mainly on oxidative stress, quercetin is an effective chelator of oxygen radicals and metals. Previous research showed that quercetin treatment can prevent chronic Cd-induced oxidative stress and renal tubular damage. In addition, quercetin has shown its efficient action in the reproductive system of animals affected by the heavy metal Cd, restoring the activity of germ cells and improving the survival of follicles damaged by Cd [127]. The above studies have verified the inhibitory effects of quercetin on Cd-induced oxidative stress and other toxic functions, and further studies have shown that the combined treatment with α-tocopherol (AT) and quercetin provided more significant protection against Cd-induced oxidative stress and lipid metabolism compared with quercetin alone, thereby reducing Cd-induced cardiovascular disease [128].

#### 3.3.2. Preventing Iron-Induced Toxic Diseases by Quercetin

Iron is an essential nutrient for the body and its deficiency affects many normal physiological functions; however, iron overload has been found to be associated with various human diseases, with diabetes being the most typical and the fastest growing worldwide [129,130]. The number of people with diabetes is increasing every year, with type 2 diabetes mellitus accounting for most cases of diabetes (about 90%), which places a heavy burden on the world economy and health systems [131]. Abnormal iron status was observed in many cross-sections in type 2 diabetes [132], and iron deposition in the islets is higher in the presence of type 2 diabetes [133]. In addition, iron sagging is a newly discovered form of regulated cell death characterized by irreparable lipid peroxidation due to overproduction of ROS in an iron-dependent manner, which has been shown to play a fundamental pathological role in various diseases associated with iron overload or dysfunction [134,135]. In contrast, there is a potential beneficial effect of quercetin on iron sagging caused by iron overload and consequently a range of diseases [136]. Lesjak [137] confirmed that quercetin can regulate iron homeostasis in the presence of iron overload. The unique properties of novel iron oxide nanoparticles (IONPs) as targeting carriers have been reported to make them suitable biomaterials for medical applications [138]. Although the advent of IONPs has provided additional convenience to medical technology, in vivo and in vitro studies have provided evidence of the possible neurotoxicity of IONPs due to free iron accumulation, ROS production, and protein aggregation [139,140,141]. Quercetin counteracts iron loading through iron chelating activity, iron homeostasis gene regulation, the attenuation of the Fenton/Haber–Weiss reaction and free radical scavenging, and the inhibition of protein aggregation [142].

#### 3.3.3. Preventing Lead-Induced Toxic Diseases

When Pb enters the body, it harms several systems, such as neurohematopoietic, digestive and renal, and cardiovascular and endocrine systems, inducing cognitive impairment and neuronal degeneration [143]. Most of the common cases of lead poisoning today involve mild chronic lead poisoning, and the main lesions are the effects of lead on metal ions and enzyme systems in the body, which cause plant nervous disorders, anemia, and immune deficiency. One study administered lead acetate intravenously to mice immediately following daily intravenous injections and proved that quercetin reduced the oxidative burden on the brain induced by lead poisoning and inhibited Pb-induced neurotoxicity in a dose-dependent manner, thereby maintaining normal physiological function in lead-poisoned mice [144,145].

## 4. Summary and Prospects

Quercetin has shown good therapeutic activity against various diseases in previous research and practice. Its powerful oxidative properties and biological activity hold great promise for clinical applications: quercetin is a natural and safe antioxidant with minimal side effects, which can be widely applied in medicine and animal feed. Quercetin exerts a good blocking effect on various toxic diseases discussed in this review and is expected to be a new drug that can prevent and treat various toxic diseases.

In addition, the therapeutic potential of quercetin against COVID-19 was found in studies by Ruben et al. and Derosa et al. [146,147]. Quercetin has shown broad antiviral properties that interfere with multiple steps of pathogen virulence-viral entry, viral assistance, and protein assembly was found to potentiate these therapeutic effects by combining with vitamin C [146].

For autoimmune diseases, which are inherently incurable systemic diseases such that patients are unable to eradicate the disease despite long-term treatment, quercetin was predicted to afford a potential opportunity and complement to the treatment and prevention of autoimmune diseases in a study by Shen et al. [148].

Among the impeding effects of quercetin on cancers, ovarian cancer is the dominant gynecological tumor due to its serious threat to women with multiple molecular mechanisms of etiology, specifically inflammation, oxidative stress, and DNA damage. A study by Tang and Shafabakhsh et al. [149,150] confirmed that quercetin can exert anti-cancer effects by inhibiting cell proliferation, promoting apoptosis, altering cell cycle progression, and affecting autophagy.

Work related to quercetin and its application in disease treatment or therapy remains sparse, and most research therein focuses on non-modified quercetin (agarose form), but the effects of the basic form of quercetin (dietary quercetin glycosides) and its effects remain to be investigated.

## Figures and Tables

**Table 1:** Toxic effects of different fungal toxins and protective effects and mechanisms of quercetin.

Fungi	*Fusarium*		*Aspergillus flavus*, *Aspergillus parasiticus*	*Aspergillus ochratoxin*, *Aspergillussulphureus*
**Mycotoxins**	Deoxynivalenol (DON)	Zearalenone (ZEN)	Aflatoxin B1 (AFB1)	Ochratoxin A (OTA)
**Structure**	^ 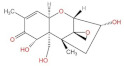 ^	^ 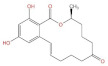 ^	^ 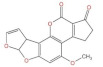 ^	^ 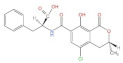 ^
**Main area of action**	Gastrointestinal and immune system	Reproductive organs	Liver, spleen, kidney	Liver, kidney
**Main toxic effects**	Fine cell and body fluid mediated, inhibition of protein synthesis	Lipid peroxidation, mitochondrial pathway, and DNA damage	Mutagenic, deformogenic, and carcinogenic	Hepatotoxicity, nephrotoxicity, teratogenicity, and immunosuppression
**Protective effect of quercetin**	Protects caco-2 cells from damage	Protects HEK293 and HCT116 cells and inhibits apoptosis	Improves the brain, enhances learning and memory, inhibits biotransformation of AFB1, and delays degenerative neurological diseases	Protects cells from damage
**Protective mechanism of quercetin**	Inhibits the production of ROS and increases cellular activity	Inhibits ROS production, antioxidant activity, and reduces ER256 levels	Increases GSH levels, competes with AFB1 for binding sites, increases glutathione peroxidase levels, increases oxidative dismutase activity, and reduces lipid peroxidation reaction	Activation of PI3K/AKT signaling pathway and reduction of ROS levels
**References**	[24,25,35]	[54,55,56,57]	[37,39,40,42,44]	[50,58]
